# Ethnic and Gender Differences in Family Social Support among Black Adolescents

**DOI:** 10.3390/healthcare6010020

**Published:** 2018-03-02

**Authors:** Christina J. Cross, Robert Joseph Taylor, Linda M. Chatters

**Affiliations:** 1 Department of Sociology, Gerald R. Ford School of Public Policy, University of Michigan, Ann Arbor, MI 48109, USA; 2 School of Social Work, Institute for Social Research, University of Michigan, Ann Arbor, MI 48109, USA; rjtaylor@umich.edu; 3 School of Public Health, School of Social Work, Institute for Social Research, University of Michigan, Ann Arbor, MI 48109, USA; chatters@umich.edu

**Keywords:** extended family, social support, support networks, race/ethnicity, adolescence

## Abstract

This study examines black adolescents’ reports of the most helpful types of social support that they receive from and provide to family members, and whether family support exchanges vary by ethnicity (African American vs. Black Caribbean) and gender. Data for this study are from the National Survey of American Life Adolescent Supplement (NSAL-A), a national, probability sample of African American and Black Caribbean youth (ages 13–17). Overall, youth reported financial support, followed by emotional assistance and practical support as the most helpful types of support that they received. Practical and emotional assistance characterized the most commonly reported types of support that they provided to family members. Black Caribbean adolescents were more likely than African American adolescents to report financial and practical assistance as the most helpful types of support that they received from family members; no ethnic differences were observed in the provision of support to relatives. There were no significant gender differences in the receipt of support, but adolescent girls reported greater involvement in providing emotional support and caregiving than adolescent boys. The results of this paper reveal that African American and Black Caribbean adolescents are involved in a complex pattern of reciprocal support exchanges with their extended family members. Study findings also reinforce the importance of research focused on racial/ethnic and gender differences in family support exchanges in order to develop a more nuanced understanding of family support behaviors within these groups.

## 1. Introduction

Social support from family members plays a key role in the wellbeing and development of adolescents [1]. Family social support has been known to help shape adolescents’ personal competencies, self-concept, and self-esteem [1,2,3], facilitate social and emotional adjustment [4,5,6,7,8], and aid in developing positive racial/ethnic identities among minority youth [9,10]. Despite increased recognition of family social support for youth developmental outcomes, few studies focus on ethnic minorities. Further, research on black adolescents and within-group heterogeneity for this population, is especially limited. Consequently, little is known about how family members are most helpful to black adolescents, and the ways in which youth, in turn, are most helpful to their family members.

This study seeks to address these gaps by examining black adolescents’ reports of social support exchanged with family (both received and provided) and the extent to which family support exchanges vary by ethnicity (African American vs. Black Caribbean) and gender. The literature review begins with an overview of social exchange theory as a theoretical framework for this study, followed by a review of research on African American and Black Caribbean family social support. This section concludes by describing the focus of the present investigation and proposing relevant hypotheses regarding ethnic, gender and sociodemographic differences in adolescent family support exchanges.

### 1.1. Social Exchange Theory

Social exchange theory (SET) posits that social behavior and interactions among individuals are a result of an exchange process; that is, a series of interdependent transactions that generate obligations and a sense of interpersonal attachment [11,12,13]. SET theorists maintain that certain rules and norms of exchange are “the guidelines” of exchange processes, and reciprocity or repayment in-kind is widely considered the fundamental principle of exchange [11,13]. Many scholars assert that how reciprocity is construed and achieved depends, in part, on the nature of the social relationship between exchange partners. Relationships that are short-term and less emotionally close typically require immediate, in-kind reciprocity for support exchanges. In long-term and close-knit relationships, however, reciprocity may emerge over an extended period of time and involve comparable, but not necessarily identical forms of exchanges [13,14]. An individual might expect swift and exact repayment for money borrowed by a co-worker but may accept a more delayed and potentially different form of repayment from a family member. Thus, SET emphasizes the importance of reciprocity in social exchanges including, but not limited to, family social support.

Further, SET maintains that the nature of the relationship between exchange partners, as well as sociodemographic characteristics such as race/ethnicity and gender, are important in shaping the form that reciprocity takes [13]. With regard to race/ethnicity, research indicates that Black Americans, as compared to Black Caribbeans are more frequently in contact with and more likely to have daily interaction with family members [15], likely because of high levels of geographic dispersion among Black Caribbean families [16,17]. They are also more involved with congregation support networks than Black Caribbeans, and report receiving higher levels of emotional support from congregation members [15]. With respect to gender, women, are more likely than men to exchange emotional support with family members and to provide more child care and care to ill family members [13,18]. Demonstrated gendered differences in social networks indicate that women have more extensive social ties than men, are socialized at an early age to fulfill gender-specific functional roles (e.g., caregiving and household work), and are more involved in emotional work (e.g., kin-keeping) within their families [19,20].

### 1.2. African American and Black Caribbean Family Social Support

Prior research indicates that African American and Black Caribbean families engage in complex webs of support exchange that function to redistribute resources and reduce economic risks [21,22,23,24,25,26]. Family support networks operate with informal norms and expectations of reciprocity among members and involve exchanges of substantial amounts of emotional, practical, and financial support [21,27,28,29,30,31]. The few studies focusing specifically on black adolescents indicate that youths report receiving high levels of emotional support, parental supervision, and discipline from family members [32,33,34]. Further, black youth also report providing various forms of practical and emotional assistance to family members, such as help with household chores, childcare, and emotional support [9,26,35,36]. Gender differences in black adolescents’ reports of family social support, indicate that adolescent boys are more likely to report greater levels of support from male family members (e.g., fathers or uncles) than adolescent girls [34,37,38]. Further, recent research examining ethnic differences in family support networks documents that African American and Black Caribbean families have distinctive patterns of kin support involvement [30,39,40].

### 1.3. Focus of the Present Investigation

This study seeks to add to the literature on the family social support networks of black adolescents by identifying the most helpful types of support that are received and provided by these youths, and whether family support exchanges vary by ethnicity and gender. Given the documented importance of family supports for adolescent development, understanding the types of family support that black youth exchange within their family systems can better inform family policies and practices that promote adolescent adjustment and wellbeing.

## 2. Methods

### 2.1. Sample

This analysis is based on data from the National Survey of American Life Adolescent sample (NSAL-A), a supplemental study of adolescents who were attached to adult households from the National Survey of American Life (NSAL). The NSAL is a stratified, multistage area probability sample of 3570 African Americans, 1621 Black Caribbeans, and 891 non-Hispanic White adults that was collected by the Program for Research on Black Americans at the University of Michigan’s Institute for Social Research. Data collection took place between February 2001 and June 2003, and the study focuses on social support, psychological and environmental stressors, and mental health disorders (see [41,42], for more detailed information about the NSAL and NSAL-A).

The adolescent sample of the 2001–2003 NSAL was drawn from the African American and Black Caribbean households only. Every household that included an adult participant in the NSAL was screened for an eligible adolescent (13–17 years of age) living in the household; adolescents were then selected using a randomized procedure. If more than one adolescent in the household was eligible, up to two adolescents were selected for the study, and if possible, the second adolescent was of a different gender [42]. A total of 1170 face-to-face interviews were conducted, including 810 African American and 360 Black Caribbean adolescents. Interview questions asked respondents about their demographic characteristics, employment, school activities, religious beliefs, psychological wellbeing, and various forms of social support. NSAL-A survey weights were designed to adjust for variation in probabilities of selection within households, and nonresponse rates for adolescents and households. The weighted data were poststratified to approximate the national population distributions for gender (male and female subjects) and age (13-, 14-, 15-, 16-, and 17-year-old) subgroups among black youth [42]. The weighting process allows us to make accurate inferences about the national population of black youth. Data collection for the NSAL-A was approved by the University of Michigan Institutional Review Board; the data is available through the Inter-University Consortium of Political and Social Research at the University of Michigan.

### 2.2. Measures

#### 2.2.1. Dependent Variable

There are two main dependent variables in this analysis: The most important type of support received from extended family members and the most important type of support given to extended family members. The most important type of support received was derived from two questions. First respondents were asked: “How often do people in your family—including parents, grandparents, brothers and sisters, aunts, uncles, cousins and so on—help you out? Would you say very often, fairly often, not too often, or never?” Respondents who reported receiving assistance were additionally asked this open-ended question: “In what way are they most helpful to you?” Adolescents’ first response was recorded and coded into broader categories based on content similarity (e.g., financial assistance, transportation, advice, assistance with homework). These categories make up the variable most important type of support received.

The most important type of support provided by adolescents to extended family members was derived from two similar questions. Respondents were first asked: “How often do you help out people in your family—including parents, brothers and sisters, grandparents, aunts, uncles, cousins and so on? Would you say very often, fairly often, not too often, or never? Respondents who indicated that they provided assistance were then asked the open-ended question: “In what ways are you most helpful?” Adolescents’ first response was recorded and coded into broader categories based on content similarity (e.g., help with chores/work/errands, help with child care/elder care). These categories make up the variable, most important type of support provided.

Bivariate analysis was conducted on the all of the categories of the most important type of support received and the most important type of support provided. Multivariate analyses were conducted on responses that had 100 or more cases.

#### 2.2.2. Independent Variables

Ethnicity, age, gender, and income are variables of interest that are used to explore possible differences in the most helpful forms of support black adolescents report receiving and giving to family. Ethnicity is a binary variable indicating whether the respondent is African American (reference) or Black Caribbean. To determine ethnicity, respondents first self-identified their race as Black. Individuals were then coded as Black Caribbean if they (a) answered affirmatively that they were of West Indian or Caribbean descent; (b) said they were from a country included on a list of Caribbean area countries presented by the interviewers, or (c) indicated that their parents or grandparents were born in a Caribbean area country. Age is coded in years and gender is a binary variable specifying whether the respondent is male (reference) or female. Family income is coded in dollars.

#### 2.2.3. Analysis Strategy

We conducted descriptive analyses to document the most useful types of support that adolescents provided to and received from family members and to describe the demographic characteristics of the sample. The Rao-Scott chi-square statistic, a complex design-corrected measure of association, was used to test for whether the types of support reported as most helpful by adolescents differed by ethnicity and gender. Bivariate analysis was conducted on all mentions of the most important type of assistance except when the number of cases was too small. Logistic regression analysis was conducted on all mentions of the most important type of support received or given which had at least 100 cases. Logistic regression analyses assessed whether any observed ethnic and/or gender differences remained after taking into account two important demographic factors which have known associations with support exchanges, age and family income. Computations for the distribution of the sociodemographic characteristics and chi-square and logistic regression analyses were conducted using SAS 9.1.3 (SAS Institute Inc., Cary, NC, USA), which uses the Taylor expansion approximation technique for calculating the complex design-based estimates of variance. All analyses utilize sampling weights to account for the complex multistage clustered design of the NSAL-A sample, unequal probabilities of selection, and post-stratification to produce nationally representative population estimates and standard errors that are generalizable to the African American and Black Caribbean adolescent populations.

## 3. Results

### 3.1. Description of Sample

Overall, African Americans make up the majority of the sample (69%), and Black Caribbeans comprise (31%) of sample members. In both the African American and Black Caribbean samples, the average respondent age is 15 years, and both groups are roughly evenly split between male and female participants. African American adolescents have average family incomes that are slightly lower than their Black Caribbean peers, with annual reported incomes of $38,292 and $38,830, respectively.

### 3.2. Receiving Support

Table 1 presents analysis of ethnic and gender differences in the most helpful type of support received from family members. Overall, “finances, money, getting needed things” was the most frequently reported form of support received by adolescents (*n* = 220), followed by “direction, advice, teaching right from wrong” (*n* = 185), and help with “school, homework” (*n* = 182). Combined, these three categories (*n* = 587) represent approximately 50% of all participants’ responses. In contrast, “not causing worry/trouble; being obedient; good grades” (*n* = 1), help with “sports” (*n* = 3), and help “when sick/injured” (*n* = 3) were the least frequently reported forms of support. Taking into account ethnicity, there were no bivariate statistically significant differences in the types of support received by African American and Black Caribbean adolescents. However, there were a couple of statistically significant gender differences in the receipt of support. Adolescent girls in comparison to adolescent boys more frequently reported that “direction, advice, teaching right from wrong” or “can talk to them when in trouble/have problems” were the most helpful support they received from extended family members.

Table 2 displays odds ratios from logistic regression analyses on ethnic and gender differences in the largest categories reported as the most helpful type of support received from family members. When we include demographic controls for age and income in our logistic regressions, the gender differences that we observe in bivariate analysis of the receipt of support are no longer significant. We do note a couple of differences by ethnicity. Black Caribbeans are more likely to report that help with “finances, money” and “when in trouble, having problems” are the most helpful types of support that they receive, relative to their African American counterparts. Additionally, we see that older adolescents, compared to younger adolescents are less likely to report help with “school, homework” as the most helpful type of support received, and they are more likely to report that assistance with “finances, money” is the most helpful type of support received. Further, adolescents from higher income households are less likely to name help with “finances, money” as the most helpful form of support they receive and are more likely to identify “advice, help with moral direction” as the most helpful type of support that they received, relative to adolescents from lower income families.

### 3.3. Providing Support

Table 3 shows analysis of ethnic and gender differences in the most helpful type of support provided by adolescents, “help with chores/work/errands” was by far the most frequently reported *n* = 431), followed by “help with child care/elder care” (*n* = 124), and “support; love; caring; comfort” (*n* = 74). Together, these three categories (*n* = 635) characterize approximately 60% of all participants’ responses. The least common types of helpful support reported were “basics (food, clothing and shelter)” (*n* = 1), help with “sports” (*n* = 1), and “take care of me/them; good job of parenting” (*n* = 4). Again, we observe no statistically significant ethnic differences in the provision of most helpful type of support. Adolescent girls, however, differed from adolescent boys in several types of support that they more frequently provided to family members: (1) “direction, advice, teaching right from wrong”; (2) “getting needed things”; (3) “anything/everything; always there for them”; (4) “can talk to me”; (5) “when in trouble/have problems”, and (6) help with child care/elder care. Adolescent boys indicated that they helped with “chores/work/errands” and provided “finances, money and obtained needed things” more frequently than adolescent girls.

Table 4 reports odds ratios from logistic regression models on the most frequently reported most helpful type of support provided to family members. When we take into account age and income, we observe that adolescent girls are still substantially (2.5 times) more likely to report assistance with “child care, elder care” as the most helpful type of support that they provide, relative to adolescent boys. Additionally, older youths are less likely to report help with “chores, errands” as the most helpful type of support provided, compared to their younger counterparts.

## 4. Discussion

This study examined the most helpful types of support that black adolescents received from and provided to extended family members, and whether these support exchanges varied across ethnicity and gender. Overall, we see that African American and Black Caribbean youth reported financial support (i.e., “finances, money, getting needed things”), followed by emotional assistance (i.e., “direction, advice, teaching right from wrong”) and practical support (i.e., help with “school/homework”) as the most helpful types of support that they received. Practical (i.e., “help with chores/work/errands” and “help with child care/elder care”) and emotional assistance (i.e., support; love; caring; comfort) characterized the most commonly reported types of support that these youths provided to family members.

These findings suggest that while black adolescents engage in reciprocal support exchanges with family members, the types of reciprocated support may be different. That is to say, the type of support adolescents receive from family members is often different from what they provide to family members. Sarkisian and Gerstel (2004) [4] refer to support exchanges of this type as involving generalized reciprocity in which there is no strict obligation for an exchange of the same type of support. Rather, participants provide forms of help and support, depending on their ability and available resources. Given that most adolescents have limited financial resources, it is not surprising that they report financial assistance as the most helpful type of support that they receive from family members. In contrast, financial assistance was not often mentioned as one of the most helpful types of support that they provide to family members. Further, this form of reciprocity affirms Social Exchange Theory’s proposition that reciprocity is shaped by relationship characteristics. Specifically, because most family relationships are characterized by emotional closeness and long-term duration, identical forms of support reciprocity are not necessarily expected.

With regard to ethnic differences in the most helpful types of support, results from chi-square analysis indicate that African American and Black Caribbean adolescents received and provided similar types of support from family members. Further analysis from logistic regression models reveal, however, two ethnic differences in the type of most helpful support received. Black Caribbean adolescents were more likely to report assistance with “finances, money” and “when in trouble, have problems” as the most helpful type of support that their family members give them. Similar to the chi-square results, no ethnic differences were observed in the logistic regression analysis of most helpful type of support provided to relatives. These findings depart from previous research focused on family social support among adults (18 years and older), which indicates distinct ethnic differences in patterns of both receiving and giving [30]. By way of explanation, African American and Black Caribbean adolescents are more similar to one another with regard to age, family and life circumstances, and resources than are their adult counterparts. Given this, they are similarly constrained in the types of support that they are able to provide, and for the most part, in the types of support they find most useful. The two noted differences in support received may reflect ethnic differences in economic resources for these groups. Black Caribbeans have higher average household incomes than African Americans [43], which may mean that they are able to provide more financial assistance to adolescents, especially when they are “in trouble, have problems”.

Taken together, these findings provide a more nuanced portrayal of the amounts and types of support adolescent girls and boys exchange with family members. Results from chi-square tests of association show several differences in family social support among subgroups of black youth. Adolescent girls were more likely than adolescent boys to report receiving emotional support (e.g., “can talk to them”) from family members. Boys, on the other hand, indicated receiving direction, advice, and teachings about right and wrong from their family members. In our logistic regression analysis, however, these gender differences no longer appear significant. In terms of providing support to family members, given their age, resources, and life circumstances, it might be expected that adolescents’ ability and expectations to provide assistance to family members would be limited. Somewhat surprisingly, our chi-square analyses demonstrate that adolescent girls and boys provided several forms of practical, emotional, and financial support to family members. Assistance with household chores and family caregiving were the most numerous categories of support provided by adolescents. Adolescent girls were more likely than boys to report that they provide emotional support and help with problems (e.g., “can talk to me”), moral instruction (e.g., “direction, advice, and teachings about right and wrong”), and various forms of practical support (e.g., “help with child care/elder care”) to family members. Adolescent boys were involved to a greater extent in providing finances and money, “help with chores, work or errands”, and providing comprehensive supports (e.g., “anything/everything”). When we take into account income and age in our logistic regression analysis, we find that gender differences in “help with chores, work, or errands” are no longer significant, but differences in “child care, elder care” remain highly significant.

These patterns of giving family support are consistent with studies of gender-role socialization within families. Girls were involved to a greater extent than boys in providing emotional support and caregiving. Multiple studies document that parents raise children to engage in gender-specific interaction styles and activities in their families. Girls are encouraged to be more emotionally expressive than boys [19,20,44], findings that align with research on women’s roles as “kin keepers” within extended family networks. In a similar vein, women tend to be more socially integrated within their extended family networks, have more frequent contact with family members, and provide help to sick relatives [29,45,46,47,48].

All in all, results of this study demonstrate the existence of certain within-race heterogeneities in the type and frequency of family social support received and provided by black adolescents. These findings may help explain the differential impact of family support on various health outcomes for subgroups of black youth. For instance, recent research finds that while family social support is not a protective factor against obesity for African American and Black Caribbean boys and for girls overall, it is associated with lower risk of obesity for African American girls [49]. On the other hand, family support is related to lower risk of depressive symptoms for African American boys, compared to African American girls [50]. Thus, because the receipt and provision of family social support operates differently for subgroups of black youth, we might expect to observe within-group differences in their impact on health outcomes. Therefore, while several key studies show that enhancing family social support is an effective strategy for promoting the health and behaviors of black adolescents (for examples see [51,52]), future work should consider how differences in the type and frequency of family support contribute to differential outcomes for subgroups of black youth.

## 5. Limitations

This study is not without its limitations. First, due to the descriptive focus of this study and the cross-sectional nature of the NSAL-A, we are unable to make causal inferences about the relationships between sociodemographic correlates and family support behaviors among black adolescents. Second, owing to small cell sizes, the study is not able to examine group variation (e.g., country of origin) within this group of Black Caribbean youth. Although not ideal, this study provides substantial strength in exploring distinctions between African American and Black Caribbean adolescents as an initial step in understanding within-group ethnic differences for the black adolescent population. Third, both measures of family support were self-reported and are subject to recall and social desirability biases.

## 6. Conclusions

Despite these limitations, this research contributes to literature on adolescents and family social support in several ways. The study’s use of a nationally representative sample of black adolescents provides a unique opportunity to examine gender and ethnicity differences in the family social support networks of black youth. It sheds light on the most important ways in which adolescents can contribute to and benefit from their family systems, and highlights similarities and differences across ethnic background and gender. Study findings identify both the types of support that many African Americans and Black Caribbeans receive such as financial assistance, as well as forms of aid that are infrequently noted as being the most important (e.g., providing hair care and grooming likely for younger siblings). Further, these findings reinforce the importance of research focused on racial/ethnic and gender differences in family support exchanges for developing a more nuanced understanding of family support behaviors within these groups.

## Figures and Tables

**Table 1:** Types of support African American adolescents and Black Caribbean adolescents receive from extended family members ^a^.

Types of Support	Total	African American Boys	African American Girls	Black Caribbean Boys	Black Caribbean Girls	Ethnicity	Gender	Overall ^b^
	*N*	% (*n*)	% (*n*)	% (*n*)	% (*n*)	*X*^2^	*X*^2^	*X*^2^
Finances, money, getting needed things	220	29.55 (65)	37.73 (83)	14.09 (31)	18.64 (41)	0.26	1.53	1.73
Direction, advice, Teaching right from wrong	185	38.92 (72)	31.35 (58)	16.76 (31)	12.97 (24)	0.26	6.96 **	6.62
School, homework	182	33.52 (61)	31.32 (57)	14.84 (27)	20.33 (37)	1.50	0.08	2.61
Support; love; caring; comfort	102	35.29 (36)	35.29 (36)	8.82 (9)	20.59 (21)	0.19	0.48	3.35
When in trouble/have problems	100	28.00 (28)	39.00 (39)	11.00 (11)	22.00 (22)	0.15	3.08	3.58
Anything and everything; always there for me/them	86	33.72 (29)	31.40 (27)	12.79 (11)	22.09 (19)	0.55	0.03	2.03
Basics (food, clothing and shelter)	79	31.65 (25)	40.51(32)	16.46 (13)	11.39 (9)	0.47	0.02	2.48
Can talk to them/me	52	15.38 (8)	46.15 (24)	17.31 (9)	21.15 (11)	1.30	4.71 *	7.70
Take care of me/them; good job of parenting	24	45.83 (11)	33.33 (8)	8.33 (2)	12.50 (3)	N/A	N/A	N/A
Help with chores/work/errands	23	56.52 (13)	26.09 (6)	17.39 (4)	0	N/A	N/A	N/A
Transportation	22	40.91 (9)	27.27 (6)	22.73 (5)	9.09 (2)	0.00	N/A	2.83
Religious support	14	21.43 (3)	50.00 (7)	21.43 (3)	7.14 (1)	N/A	N/A	N/A
Help with child care/elder care	7	0	100 (7)	0	0	N/A	N/A	N/A
Do things, help do things	6	33.33 (2)	66.67 (4)	0	0	N/A	N/A	N/A
Help when sick/injured	3	0	100 (3)	0	0	N/A	N/A	N/A
Sports	3	100 (3)	0	0	0	N/A	N/A	N/A
Not causing worry/trouble; being obedient; good grades	1	0	0	100 (1)	0	N/A	N/A	N/A
Total	1170	33.93 (397)	35.30 (413)	14.1 (165)	16.7 (195)			

^a^ Percentage values are weighted; frequencies are unweighted. * *p* < 0.05; ** *p* < 0.01; *** *p* < 0.001; ^b^ Overall column refers to differences by ethnicity and gender in the types of social support most frequently received by adolescents.

**Table 2:** Logistic regression analyses of the most important type of support received from extended family members among African American and Black Caribbean adolescents.

Independent Variables	Model 1	Model 2	Model 3	Model 4	Model 5
	Finances, Money	Advice, Moral Direction	School, Homework	Emotional Support, Caring, Comfort	When in Trouble, Having Problems
	OR (95% C.I.)	OR (95% C.I.)	OR (95% C.I.)	OR (95% C.I.)	OR (95% C.I.)
Ethnicity					
Black Caribbean	1.59 (1.12, 2.28) *	0.69 (0.40, 1.18)	1.13 (0.45, 2.85)	0.73 (0.26, 2.08)	1.57 (1.02, 2.41) *
Age	1.27 (1.10, 1.48) **	0.94 (0.81, 1.09)	0.80 (0.71, 0.91) **	1.08 (0.90, 1.29)	1.05 (0.89, 1.24)
Gender					
Female	1.32 (0.87, 2.00)	0.72 (0.51, 1.02)	0.89 (0.56, 1.40)	0.91 (0.56, 1.47)	1.14 (0.72, 1.81)
Household Income	0.94 (0.90, 0.99) *	1.02 (1.00, 1.04) *	1.00 (0.99, 1.01)	1.02 (1.00, 1.04)	0.98 (0.95, 1.02)
F	6.04 ***	2.62	3.20 *	1.17	2.06
Df	40	40	40	40	40
N	1115	1115	1115	1115	1115

* *p* < 0.05; ** *p* < 0.01; *** *p* < 0.001; OR = Odds Ratio; CI = Confidence Interval.

**Table 3:** Types of support African American adolescents and Black Caribbean adolescents provide to extended family members ^a^.

Types of Support	Total	African American Boys	African American Girls	Black Caribbean Boys	Black Caribbean Girls	Ethnicity	Gender	Overall ^b^
	*N*	% (*n*)	% (*n*)	% (*n*)	% (*n*)	*X*^2^	*X*^2^	*X*^2^
Help with chores/work/errands	431	36.19 (156)	30.63 (132)	16.94 (73)	16.24 (70)	2.00	8.67 **	10.41 *
Help with child care/elder care	124	22.58 (28)	46.77 (58)	6.45 (8)	24.19 (30)	0.00	19.92 ***	20.82 ***
Support; love; caring; comfort	74	33.78 (25)	37.84 (28)	17.57 (13)	10.81 (8)	0.21	0.39	2.26
Anything/everything; always there for them	71	49.30 (35)	21.13 (15)	12.68 (9)	16.90 (12)	0.05	6.06 *	10.33 *
Finances, money, getting needed things	57	49.12 (28)	31.58 (18)	12.28 (7)	7.02 (4)	3.70	4.42 *	8.10 *
Can talk to them	53	28.30 (15)	49.06 (26)	5.66 (3)	16.98 (9)	1.72	4.32 *	6.12
Direction, advice, teaching right from wrong	53	24.53 (13)	39.62 (21)	9.43 (5)	26.42 (14)	0.68	4.32 *	5.83
When sick/injured	52	26.92 (14)	42.31 (22)	13.46 (7)	17.31 (9)	0.00	1.23	1.42
When in trouble/have problems	46	21.74 (10)	50.00 (23)	8.70 (4)	19.57 (9)	0.14	5.85 *	6.04
School, homework	31	29.03 (9)	41.94 (13)	16.13 (5)	12.90 (4)	0.04	0.09	0.86
Do things, help do things	30	43.33 (13)	36.67 (11)	10.00 (3)	10.00 (3)	N/A	N/A	N/A
Electronics/technical	11	27.27 (3)	18.18 (2)	54.55 (6)	0	N/A	N/A	N/A
Not causing worry/trouble; being obedient; good grades	10	60.00 (6)	20.00 (2)	0	20.00 (2)	N/A	N/A	N/A
Good example for others; keep others from getting in trouble	9	33.33 (3)	33.33 (3)	33.33 (3)	0	N/A	N/A	N/A
Transportation	8	0	50.00 (4)	37.50 (3)	12.50 (1)	N/A	N/A	N/A
Do their hair	6	0	83.33 (5)	0	16.67 (1)	N/A	N/A	N/A
Take care of them; good job of parenting	4	50.00 (2)	0	25.00 (1)	25.00 (1)	N/A	N/A	N/A
Religious support	2	50.00 (1)	50.00 (1)	0	0	N/A	N/A	N/A
Basics (food, clothing and shelter)	1	100 (1)	0	0	0	N/A	N/A	N/A
Sports	1	0	100 (1)	0	0	N/A	N/A	N/A
Total	1080	33.93 (397)	35.30 (413)	14.1 (165)	16.7 (195)	173.1 ***	1.65	175.05 ***

^a^ Percentage values are weighted; frequencies are unweighted. * *p* < 0.05; ** *p* < 0.01; *** *p* < 0.001; ^b^ Overall column refers to differences by ethnicity and gender in the types of social support most frequently provided by adolescents.

**Table 4:** Logistic regression analyses of the most important type of support provided to extended family members among African American and Black Caribbean adolescents.

Independent Variables	Model 1	Model 2
	Chores, Errand	Child Care, Elder Care
	OR (95% C.I.)	OR (95% C.I.)
Ethnicity		
Black Caribbean	1.46 (0.60, 3.55)	1.15 (0.43, 3.06)
Age	0.84 (0.73, 0.97) *	1.11 (0.92, 1.34)
Gender		
Female	0.69 (0.48, 1.00)	2.54 (1.37, 4.69) **
Household Income	1.00 (0.99, 1.02)	0.99 (0.95, 1.03)
F	2.57	5.03 **
Df	40	40
N	1074	1074

* *p* < 0.05; ** *p* < 0.01; *** *p* < 0.001; OR = Odds Ratio; CI = Confidence Interval.
